# Ketone Bodies Inhibit the Opening of Acid-Sensing Ion Channels (ASICs) in Rat Hippocampal Excitatory Neurons *in vitro*

**DOI:** 10.3389/fneur.2019.00155

**Published:** 2019-03-12

**Authors:** Fei Zhu, Wei Shan, Qinlan Xu, Anchen Guo, Jianping Wu, Qun Wang

**Affiliations:** ^1^Department of Neurology, Beijing Tiantan Hospital, Capital Medical University, Beijing, China; ^2^National Center for Clinical Medicine of Neurological Diseases, Beijing, China; ^3^Beijing Institute for Brain Disorders, Beijing, China; ^4^Advanced Innovation Center for Human Brain Protection, Capital Medical University, Beijing, China

**Keywords:** ketone body, ketogenic diet, acid-sensing ion channels, patch clamp, neuron

## Abstract

**Objectives:** Despite the long-term efficacy of antiepileptic drug treatments, frequent attacks of drug-resistant epilepsy necessitate the development of new antiepileptic drug therapy targets. The ketogenic diet is a high-fat, low-carbohydrate diet that has been shown to be effective in treating drug-resistant epilepsy, although the mechanism is yet unclear. In the ketogenic diet, excess fat is metabolized into ketone bodies (including acetoacetic acid, β-hydroxybutyric acid, and acetone). The present study explored the effect of ketone bodies on acid-sensing ion channels and provided a theoretical basis for the study of new targets of antiepileptic drugs based on “ketone body-acid sensing ion channels.”

**Methods:** In this study, rat primary cultured hippocampal neurons were used. The effects of acetoacetic acid, β-hydroxybutyric acid, and acetone on the open state of acid-sensing ion channels of hippocampal neurons were investigated by the patch-clamp technique.

**Results:** At pH 6.0, the addition of acetoacetic acid, β-hydroxybutyric acid, and acetone in the extracellular solution markedly weakened the currents of acid-sensing ion channels. The three ketone bodies significantly inhibited the opening of the acid-sensing ion channels on the surface of the hippocampal neurons, and 92, 47, and 77%, respectively.

**Conclusions:** Ketone bodies significantly inhibit the opening of acid-sensing ion channels. However, a new target for antiepileptic drugs on acid-sensing ion channels is yet to be investigated.

## Introduction

Epilepsy is a common chronic disease of the central nervous system. The point prevalence of active epilepsy was 6.38/1,000 individuals, while the lifetime prevalence was 7.60/1,000 individuals. The annual cumulative incidence of epilepsy was 67.77/100,000 persons, while the incidence rate was 61.44/100,000 person-years (1). Approximately, 70% of the patients with epilepsy can control their seizures through pharmacological therapy, while approximately, 30% of the patients persistently exhibit drug-resistant epilepsy. Epilepsy is considered as drug-resistant seizures that persist despite the administration of two antiepileptic drugs adapted to the patient's needs, with an effective and well-tolerated dosage, either as a single agent or in combination (2). The management of drug-resistant epilepsy is a major public health concern, and the development of antiepileptic drugs is based on new targets with clinical significance.

A ketogenic diet is a high-fat, low-carbohydrate diet that is effective in treating drug-resistant epilepsy (3, 4). However, the underlying mechanism is yet unclear. Long-term ketogenic diets are not well-tolerated and often cause several adverse reactions, including growth retardation in children and an increased risk of atherosclerosis in adults (4, 5). Therefore, clarifying the mechanism of action of ketogenic diets and applying new drug research and development is crucial. The ketogenic diet involves the catabolism of ketone bodies into acetoacetic acid, β-hydroxybutyric acid, and acetone in the presence of excessive fat, and several studies suggested that an acute ketone body injection into animals inhibit seizures (6–8), however, the specific mechanism is unclear. In this study, we speculated that a correlation is established between ketone bodies and acid sensitivity and explored the effect of ketone bodies on acid-sensing ion channels. Primary culture of rat hippocampal neurons with the patch clamp technique was used to study the three types of ketone body on acid-sensing ions of the hippocampal neurons. The present study examined a new method for the treatment of drug-resistant epilepsy based on the “ketone body-acid sensing ion channel” of antiepileptic drug targets in order to provide a theoretical basis.

## Materials and Methods

### Animals

Wistar rats were purchased from Beijing Virton Li Hua Experimental Animal Technology Co., Ltd. The animals were housed at a density of five mice per cage under standard conditions (23 ± 1°C, 50 ± 5% humidity) with a 12 h light/dark cycle. Food and water were available *ad libitum*. This study was approved by the Institutional Animal Care and Use Committee of Capital Medical School Affiliated Tiantan Hospital. The hippocampal neurons were dissociated and prepared from E16-E17 rat embryos as described previously (9). When the primary neuron culture experiments were carried out, the rats were decapitated under deep anesthesia with aether to reduce the pain. The experiments were performed according to the principles outlined in the Animal Research Reporting of *In Vivo* Experiments (ARRIVE) guidelines and the Basel declaration (http://www.basel-declaration.org). The Replacement, Refinement, and Reduction (3R) of Animals in research have been considered while planning the experiments.

### Main Reagents

Lithium acetoacetate (A8509, Sigma), β-hydroxybutyrate (H6501, Sigma), acetone (270725, Sigma), poly-lysine (P4707, Sigma), L-glutamyl (1294848, sigma), trypsin (T4674, Sigma), glutamic acid (G5667, Sigma), glucose (G8270, Sigma), 50X B27 supplement (17504044, Gibco), fetal bovine serum (10099-141, Gibco), DMEM (11965-092, Gibco), Neurobasal cells (21103049, Gibco), penicillin/streptomycin (SV30010, HyClone), HEPES (sc-29097, Santa Cruz Biotechnology), phosphate-buffered saline (PBS) (SH30258, HyClone), and Paraformaldehyde (P6148, Sigma).

### Primary Neuron Culture

The bottom of the plates was coated with poly-lysine at 37°C in a 5% CO_2_ incubator overnight, and then, covered for >30 min. Subsequently, the plates were washed 2–3 times and dried. Both parts of the experimental equipment were soaked into isopropyl alcohol for 1 h. Fine tweezers, a pair of iris scissors, curved scissors, and eye scissors were used to spin off the brain tissue. Nerve tweezers, big scissors, and eye scissors were used to strip the embryonic rats. Two boxes of crushed ice were used, and the test bed was cleaned. The ice bag was placed on the test board, and alcohol was sprayed to sterilize the environment.

The heads of the embryonic rats were severed, the brain was stripped, and subsequently, placed into ice-cold PBS for 2 min. The brain was removed and placed in ice-cold PBS, and a filter was placed at the bottom so that the brain could easily slide away from the plate. The skull was peeled off and exposed, and the cortex was opened. The hippocampus was located, and the surface of the membrane was cleaned. The hippocampus was removed and placed into 10 mL ice-cold PBS in a centrifuge tube. The free seahorses were washed 1–2 times in ice-cold PBS and trypsinized at 37°C. Then, the reaction was terminated, and the samples washed 2–3 times in PBS. The samples were held for 2 min at room temperature, and the upper liquid portion was removed. A total of 1 ml DMEM was added to make a blow, suck the action to be slow, especially to try to slow down, after blowing 15 times, static for 2 min, and the cleaning liquid was collected and placed into the centrifuge tube. A volume of 1 mL DMEM was added, and the supernatant was collected, while the undigested tissue was discarded. After 4 h, the dead cells and debris were subsequently washed with DMEM, and the media was changed to neurobasal medium+2% b27+1% glutamate every 2 days.

### Immunohistochemistry (IHC)

Rats were injected with tribromoethanol (200 mg/kg, i.p.) for anesthesia, and transcardially perfused with isotonic 0.1 M phosphate buffer (pH 7.4), followed by isotonic 4% PFA. Then, the brain was fixed in 4% PFA at 4°C overnight and cryoprotected in 20–30% sucrose in 0.1 M phosphate buffer. Briefly, sections (20 μm) were fixed with 4% PFA and washed with 0.3% Triton X-100/PBS. Subsequently, the sections were incubated for 1 h in blocking serum (5% normal donkey serum in 0.2% Triton-X 100/PBS) at room temperature and then with primary antibodies (anti-ASIC1, Alomone Labs, Jerusalem, Israel; anti-Parvalbumin, Swant, Marly1, Switzerland) at 4°C overnight. Finally, the sections were washed with PBS and incubated with species-matched secondary antibodies (Alexa 488-conjugated anti-mouse IgG; Alexa 546-conjugated anti-rabbit IgG, Invitrogen) at room temperature for 1 h. The images were captured using the Evos FL auto 2 microscope (Invitrogen).

### Electrophysiology

The effect of the three ketones on the open state of the acid-sensing ion channel of rat hippocampal cultured neurons was explored. The capillary glass tube (bf150-86-10, Sutter Instruments) was used to design the recording electrode with the microelectrode drawing instrument (P97, Sutter Instruments). The electrode was filled with the fluid. The inverted microscope (IX71, push around) was used for controlling the microelectrode (MP285, Sutter Instruments), the data from the electrodes in contact with the cells were recorded, giving a negative pressure suction, and forming a G Ω sealing after a rapid capacitance compensation. Next, we continued to provide negative pressure, disrupted the cell membrane absorption, and formed the whole-cell recording mode. Subsequently, the compensation for the slow capacitance, the capacitance, and series resistance were recorded. After the voltage clamp mode set at −70 mV was stable for 5 min, the acid-sensing ion channel record was carried out under 80 mV. Data Clampfit was used for offline analysis. Outside the record fluid: 150 mM NaCl, 5 mM KCl, 1 mM CaCl_2_, 1 mM MgCl_2_, 10 mM HEPES, 10 mM glucose, the pH was adjusted to 7.4 using NaOH, and the aperture of 0.22 μm membrane filtration was maintained aseptic at 4°C. Next, 20 μm DNQX and 10 μm Bicuculline were added to the external fluid to block the alpha-amino-3-hydroxy-5-methyl-4-isoxazole propionic acid (AMPA) and γ-aminobutyric acid (GABA) currents. Inside the record fluid: 130 mM CsCl, 1.6 mM MgCl_2_, 10 mM HEPES, 5 mM EGTA, and Na_2_-ATP. CsOH is used for adjusting the pH to 7.2, the aperture of 0.22 μm membrane is filtrated aseptically, and the sample was stored at −20°C.

### Statistical Methods

SPSS19.0 software was used for data analysis. The normal distribution of the measured data is represented by mean ± standard deviation. The two independent groups with the normal distribution were compared using the *t*-test. A *P* < 0.05 indicates statistical significance.

## Results

### Spatial Expression of ASIC1 in the Rat Mice Brain

**Figure 1:**
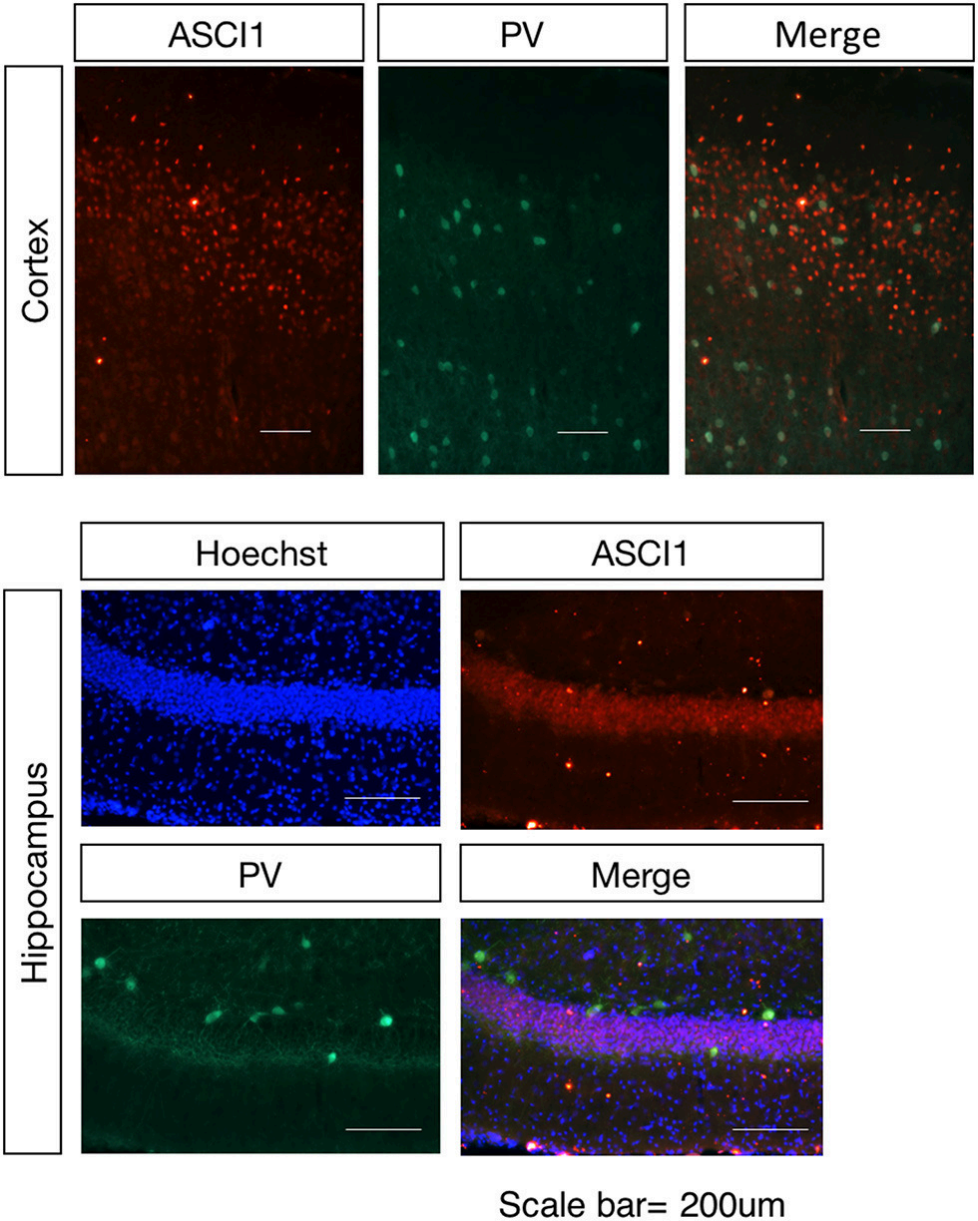
Spatial distribution of the ASIC1 protein in the cortex and hippocampus regions. Representative images show coronal sections in the cortex and hippocampus region. The expression of ASIC1 in the cortex and hippocampus in red, PV-positive neuron represented in green. Scale bars indicate 200 μm.

Based on the previous study, the ASICs in the cortex region and hippocampus region are primarily ASIC1a with limited expression of ASIC2. In order to clarify the spatial distribution of ASIC1 in the normal condition of wild-type rat and identify the target neurons for the electrophysiological assay in the next study, we investigated the expression of ASIC1 in the cortex and hippocampus of the wild-type rat in the adult stage. Double-labeling IHC revealed that strong ASIC-positive cells are not co-localized with parvalbumin (PV)-positive neurons (Figure 1), indicating that the distribution of ASIC1 is mainly in the mossy fiber neurons but limited in the non-exciting neurons, at least not in the PV-positive intern neurons in the cortex and hippocampus regions.

### Acetoacetic Acid, β-Hydroxybutyric Acid, and Acetone Significantly Inhibits the Opening of Acid-Sensing Ion Channels in Rat Hippocampal Neurons at pH 6.0

**Figure 2:**
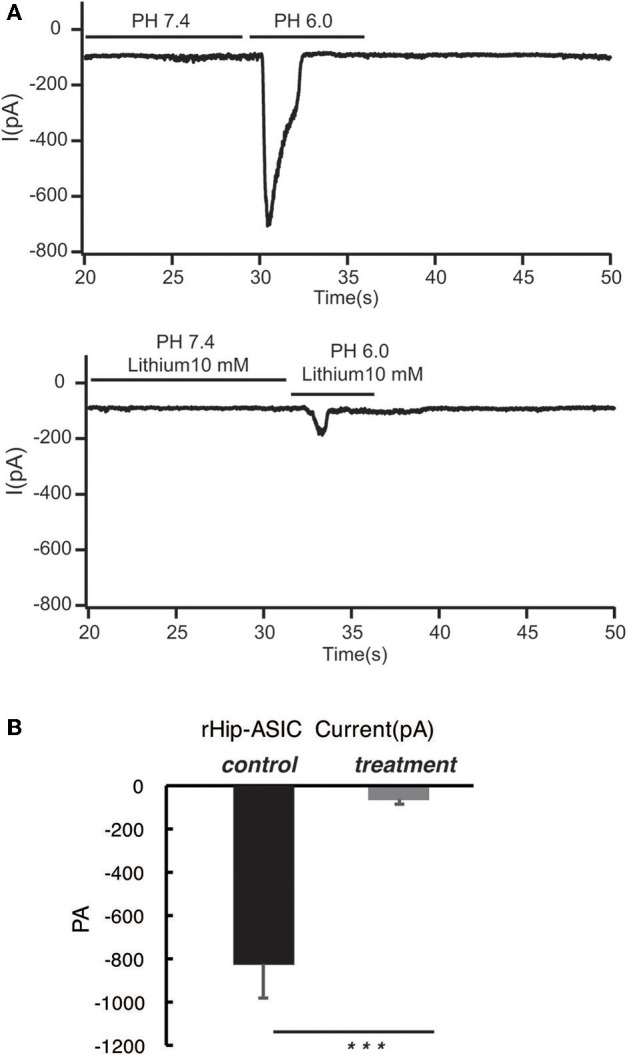
Acetoacetic acid significantly inhibits the opening of acid-sensing ion channels in rat hippocampal neurons at pH 6.0. **(A)** The current wave amplitude of the acid-sensing ion channel at pH < 6.0 was rescued by the treatment with acetoacetic acid. The upper panels show typical recordings of the current wave amplitude of the acid-sensing ion channel. **(B)** The quantification of the average amplitude of the current is shown in the lower panel. Data are presented as the mean ± SEM. The number of rats is 3–4 and that of the neurons in the test is 8–10; ***P < 0.001.

**Figure 3:**
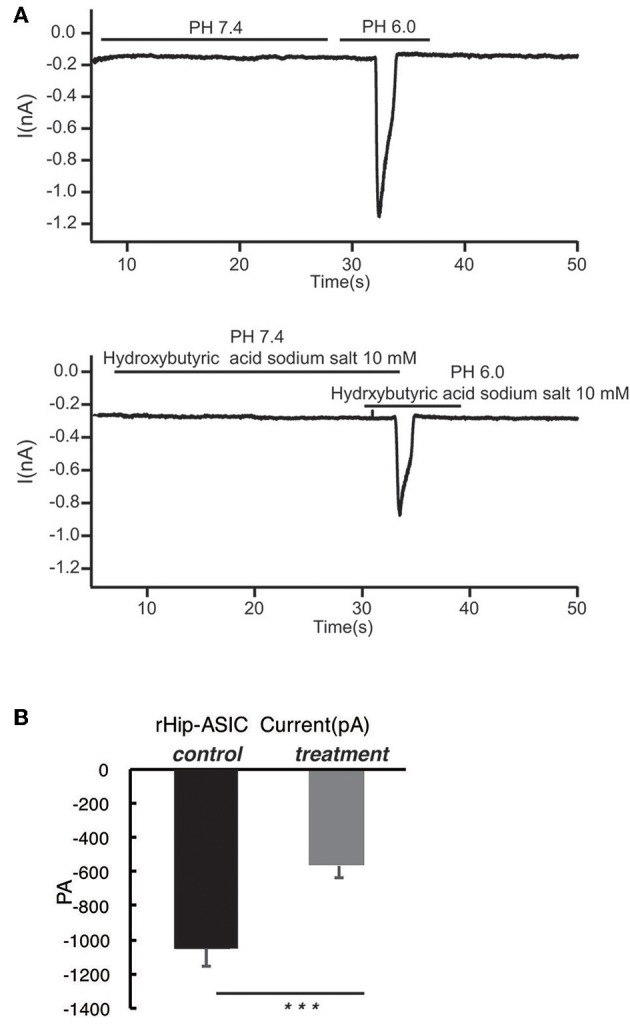
β-hydroxybutyric acid significantly inhibits the opening of acid-sensing ion channels in rat hippocampal neurons at pH 6.0. **(A)** The current wave amplitude of the acid-sensing ion channel at pH < 6.0 was rescued by the treatment with β-hydroxybutyric acid. The upper panels show typical recordings of the current wave amplitude of acid-sensing ion channel. **(B)** The quantification of the average amplitude of the current is shown in the lower panel. Data are presented as the mean ± SEM. The number of rats is 3–4 and that of the neurons in the test is 8–10; ***P < 0.001.

**Figure 4:**
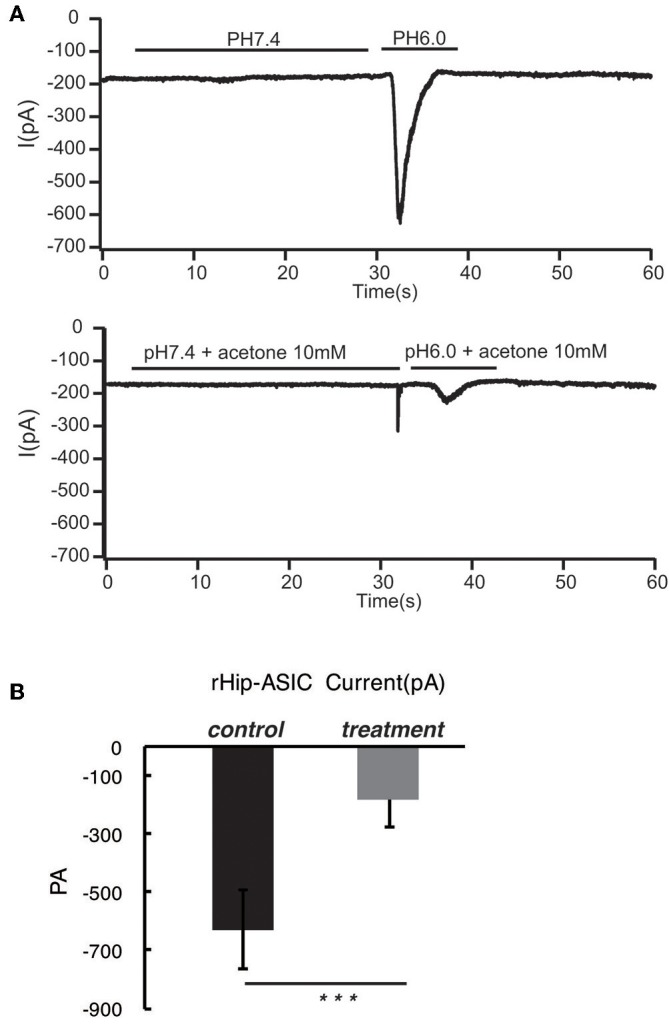
Acetone significantly inhibits the opening of acid-sensing ion channels in rat hippocampal neurons at pH 6.0. The current wave amplitude of the acid-sensing ion channel at pH < 6.0 was rescued by the treatment with acetone. **(A)** The upper panels show typical recordings of the current wave amplitude of acid-sensing ion channel. **(B)** The quantification of the average amplitude of the current is shown in the lower panel. Data are presented as mean ± SEM. The number of rats is 3–4 and that of the neurons in the test is 8–10; ***P < 0.001.

According to the previous report, ASIC1a has a pH_0.5_ of 6.2, which mediates fast decaying, transient currents. Thus, in the current study, we selected pH 6.0 to treat the hippocampus neuron such as to induce the activity of ASIC1a. Subsequently, acetoacetic acid, β-hydroxybutyric acid, and acetone were identified as potential regulatory compositions after ketogenic diets. To test the hypothesis whether these compositions can regulate the neural electrophysiology in the exciting neurons, we examined the current wave amplitude of the acid-sensing ion channel at pH < 6.0. Consequently, acetoacetic acid, β-hydroxybutyric acid, and acetone significantly decreases the current wave amplitude after treatment pH < 6.0 (Figures 2A, 3A, 4A), and the inhibitory rates were 92 ± 5% (*P* < 0.01, Figure 2B), 47 ± 7% (*P* < 0.01, Figure 3B), and 77 ± 21% (*P* < 0.01, Figure 4B) respectively, whereas no effect was detected on the basal current wave level (data not shown).

## Discussion

The main results of the present study are as follows: ASIC1 is primarily expressed in the excitatory neuron, the expression of ASIC1 in the interneuron, at least in the PV-positive neuron, is limited. The products of the ketogenic diet metabolism such as acetoacetic acid, β-hydroxybutyric acid, and acetone inhibited the acid-sensitive ion channel and its function.

The ketogenic diet is a high-fat, low-carbohydrate diet and is effective in treating drug-resistant epilepsy (10). However, the underlying mechanism is yet to be elucidated. Patients on the long-term ketogenic diet do not easily tolerate this diet, and the ketogenic diet often causes several adverse reactions, such as growth retardation in children and increased risk of atherosclerosis in adults (4, 5). Therefore, understanding the mechanism of action of ketogenic diets and applying for new drug research and development is critical.

In the ketogenic diet, excess fatty acids produce acetyl-CoA in the liver, which is then broken down into ketones, including acetic acid (ACA), β-hydroxybutyric acid (βHB), and acetone. Previous studies suggested that an acute injection into animals can inhibit the ketone body seizures (6, 7), but the specific mechanism is unclear. Moreover, ketone bodies might also function by acting on ATP-sensitive potassium channel (KATP), N-methyl-D-aspartic acid (NMDA) or γ-aminobutyric acid channels (11, 12).

In this study, we speculated a correlation between ketone bodies and acid sensitivity. Also, the effects of acetoacetic acid, β-hydroxybutyric acid, and acetone on the open state of acid-sensing ion channels of hippocampal neurons were investigated using the patch-clamp technique. We found that all the three ketone bodies significantly inhibited the opening of acid-sensing ion channels, and the rates of inhibition were 92, 47, and 77%, respectively. Thus, the ketogenic diet may be able to treat drug-resistant epilepsy through the inhibition of ketone bodies of acid-sensing ion channels.

The acid-sensing ion channels (ASICs, acid-sensing ion channels) also known as H+ gating ion channels, are a type of proton-activated cation channels, which belong to the family of element/epithelial sodium channels (degenerin/epithelial Na+ channels, DEG/ENaC). H+ is an ASIC channel agonist; therefore, ASICs can be regarded as ligands that regulate this family of channels. The ASIC family members include four genes (*Asic1, Asic2, Asic3, Asic4*). Hitherto, the six subunits of ASICs, ASIC1a, ASIC1b, ASIC2a, ASIC2b, Asic3, Asic4, have been cloned from ASICs. Tissue acidification is necessary to activate the ASICs, and the activation of ASICs depends on the rapid and significant decrease in pH (< 1 s) (13–15).

Presently, ASICs is extensively studied as major therapeutic target of acid toxicity injury (16, 17). Xiong et al. (18) demonstrated that ischemic brain injury in brain cells enhanced the anaerobic metabolism and led to a marked decline in pH. This process led to significant acidosis and ASIC1a activation, a large inflow of calcium ions, and calcium overload after extensive cell death. The addition of ASIC1a-specific blockers PcTX or the non-specific ASICs blocker amiloride *in vivo* and *in vitro* causing acidosis secondary to neuronal damage was significantly reduced, and the scope of cerebral infarction was also declined significantly. Thus, ASIC1a-mediated acidosis during cerebral ischemia resulted from neuronal death. Since the epileptic seizures, especially status epilepticus (SE), cause severe and harmful stimulation of the brain, we speculated that severe seizures might also lead to acid-sensing ion channels in the brain to mediate acid toxicity. Interestingly, because ketobodies can inhibit the opening of acid-sensing ion channels, we speculated that the ketobodies might exert a neuroprotective role by inhibiting acid-sensing ion channel-mediated brain tissue acid toxicity.

Currently, the correlation between acid-sensing ion channels and epilepsy primarily focuses on seizures after the changes in the expression of acid-sensing ion channel subunits and the antagonists on the direct effect of epileptic seizures. Wu et al. (19) found that intraperitoneal injection of the ASIC antagonist amiloride reduces the proportion of rats reaching Racine level IV seizures, protected the piriform cortex layer II and III neurons, and prolonged the survival in the acute phase following status epilepticus (SE). Using EEG studies, Liang et al. (20) observed that the intraperitoneal injection of the ASICs antagonist amiloride in rats greatly reduced the frequency of abnormal discharge within 90 min after injection. The current study found that the ASIC1a and ASIC3 expression was increased after SE. Using the patch clamp technique on hippocampal slices, Ievglevskyi et al. (21) observed that the non-selective ASIC inhibitors 5b resulted in spontaneous inhibitory postsynaptic potential discharge frequency, and the inhibition of ASICs was abated without the magnesium model and KA model-induced epilepsy discharge. In summary, non-selective ASIC receptor antagonists can suppress the seizures. Herein, we found that the ketobodies can inhibit the acid-sensing ion channels, and thus, it can be speculated that ketobodies can reduce the excitability of neurons by inhibiting the acid-sensing ion channels, thereby exerting an anti-epileptic role. However, due to the limitation of the equipment and technology, we could not test the currents under the physiological pH condition. According to the previous report (22), ASIC1a mediates fast decaying, transient currents with a pH_0.5_ of ~6.2. We followed the protocol and treated hippocampus neuron with pH 6.0 to induce the activity of ASIC1a. However, to get more solid and convincing data to support the hypothesis of this project, we acknowledge that we should test the real-time pH changes in the microenvironment and measure the neuronal currents during the epilepsy process.

Taken together, acid-sensing ion channel antagonists can inhibit seizures, acid-sensitive channels mediate the body's acid toxic effect, and ketone bodies can inhibit the acid-sensing ion channels. Thus, we speculated that ketone bodies play an antiepileptic role via two pathways. One, the ketone bodies can inhibit the acid-sensing ion channels that directly alter the nerve excitability. On the other hand, ketone bodies can inhibit an acid-sensing ion channel-mediated acid toxic effect and result in a neuroprotective effect during the epileptic seizures. Thus, the “keto-acid-sensing ion channel” seems to be a promising target for the drug-resistant epilepsy drug treatment, and hence, the underlying antiepileptic mechanism needs to be investigated further.

## Author Contributions

QW, JW, and FZ designed research. FZ, WS, AG and QX performed research. FZ and WS analyzed data and wrote the paper.

### Conflict of Interest Statement

The authors declare that the research was conducted in the absence of any commercial or financial relationships that could be construed as a potential conflict of interest.
